# Desmoid Tumor of the Rectus Abdominis Muscle: A Case Report

**DOI:** 10.7759/cureus.61383

**Published:** 2024-05-30

**Authors:** Tien Tran Phung Dung, Phuoc Tu Minh, Huan Nguyen Ngoc

**Affiliations:** 1 Digestive Surgery, Cho Ray Hospital, Ho Chi Minh City, VNM

**Keywords:** desmoid-type fibromatosis, mesenchymal tumors, rectus abdominal muscle tumor, abdominal wall tumor, desmoid

## Abstract

Desmoid tumors, also referred to as aggressive fibromatosis, represent an uncommon form of fibroblastic proliferation. These neoplasms may arise within any musculoaponeurotic structure throughout the body. They are classified as benign due to several distinctive features: histologically, they exhibit regular mitotic activity and are devoid of metastatic potential. Computed tomography (CT) remains the definitive modality for precise diagnosis, and surgical excision is strongly advised. This account details the manifestation of a desmoid tumor located in the anterior abdominal wall of a 31-year-old female patient who notably lacks any prior surgical interventions. The surgical intervention entailed the excision of the neoplasm and subsequent reconstruction of the abdominal wall utilizing a polypropylene mesh. Postoperatively, the patient was released from the medical facility after a period of three days, having experienced no post-surgical complications. This was followed by a six-month interval free of any adverse events.

## Introduction

Desmoid tumors, known as aggressive fibromatosis, constitute a scarce subset of mesenchymal neoplasms [1]. They account for a mere 0.03% of all oncological cases and 3% of soft-tissue tumors. The nomenclature 'desmoid' was coined by Muller in 1838, originating from the Greek term 'desmos', signifying a resemblance to tendons. Desmoid tumors represent monoclonal proliferations of fibroblasts within musculoaponeurotic structures. Despite their benign nature, they exhibit aggressive behavior and are of mesenchymal derivation. These neoplasms comprise a diverse array of pathological entities, characterized by the proliferation of well-differentiated fibroblasts [2,3]. Diagnostic imaging modalities such as computed tomography (CT) and magnetic resonance imaging (MRI) are instrumental in refining the differential diagnosis. Owing to the imaging resemblance among lesions, the procurement of microscopic evidence is essential for establishing a conclusive diagnosis [4]. Abdominal wall desmoid tumors concurrent with pregnancy or trauma represent an exceptionally common phenomenon. Desmoid tumors are still present in men; the tumors are twice as common in females as in males. Despite their propensity for local dissemination through the infiltration of surrounding tissues and structures, these tumors do not progress to metastatic stages. They predominantly arise in individuals within the 25-40 age bracket, exhibiting a significant incidence rate in women during their reproductive years [1].

## Case presentation

The patient presented with a complaint of an asymptomatic mass located in the upper right quadrant of her abdomen. She reported that the dimensions of the mass had remained stable since its initial discovery four months prior. The patient, a 31-year-old female, reported no consumption of tobacco or alcohol and lacked a notable familial history of associated disorders such as Gardner syndrome or familial adenomatous polyposis syndrome. Additionally, she was not undergoing any pharmacological treatments. Her obstetric history included two unassisted vaginal deliveries, with the most recent childbirth occurring four years prior. The patient's medical history was devoid of any traumatic incidents or surgical procedures.

**Figure 1:**
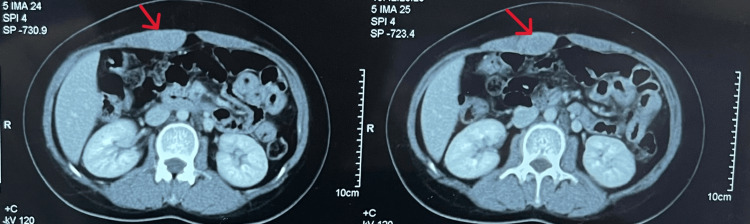
A contrast-enhanced CT scan shows a mass within the right rectus sheath

The patient's laboratory investigations did not reveal any abnormalities. A physical examination disclosed a firm, palpable mass, measuring 3 x 4 cm, adherent to the abdominal wall. Subsequent to the intravenous administration of a contrast agent, imaging studies, including CT and ultrasound, identified a hypodense lesion within the right rectus muscle, exhibiting slight enhancement on delayed phase imaging (Figure 1). In this case, we decided to remove the tumor without a core needle biopsy because the clinical presentation of the tumor and the results from the CT scan are extremely helpful in narrowing the differential diagnosis, specifically the size and depth of the lesion. Most abdominal wall desmoid tumors are benign and have no metastasis. Surgery is the mainstay of treatment in the management of desmoid tumors, and resection of abdominal wall tumors, in particular, can be performed safely.

**Figure 2:**
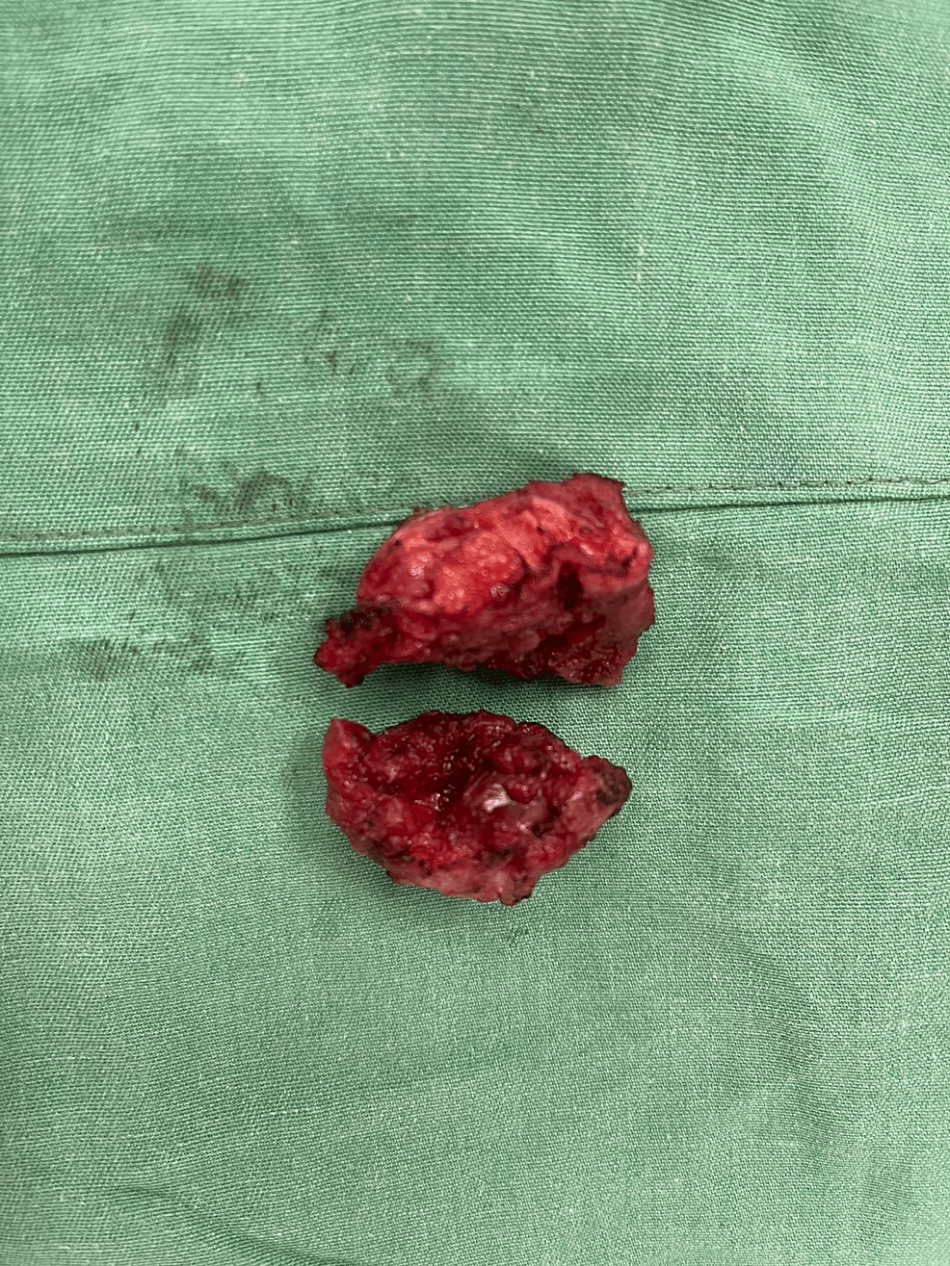
The gross specimen macroscopically demonstrates a well-defined mass measuring 3 x 4 cm

**Figure 3:**
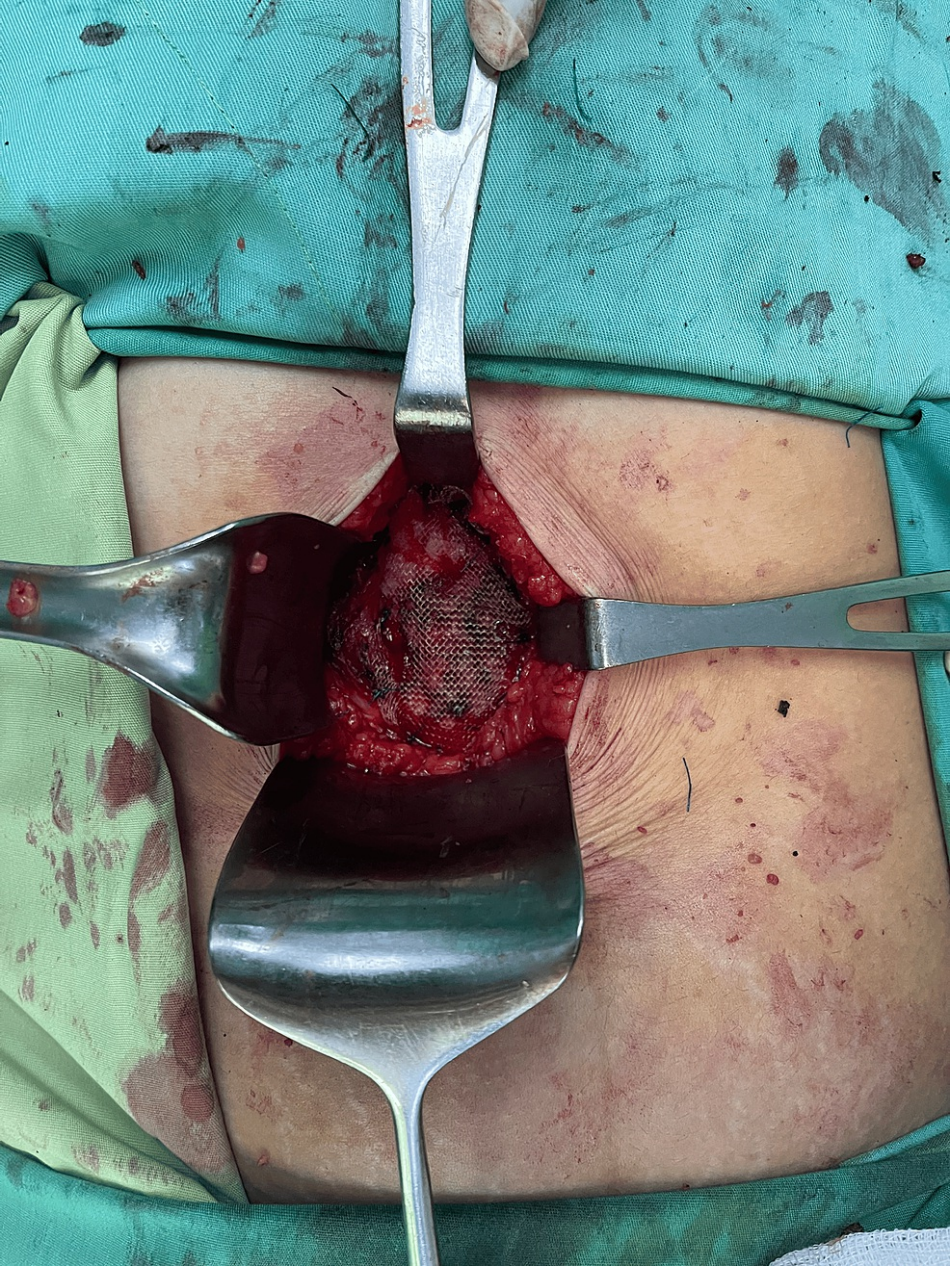
Reconstruction the abdominal wall with a propylene mesh

Administered under general anesthesia, the tumor was completely excised, preserving a margin of uninvolved muscular tissue (Figure 2). The mass, situated within the right rectus muscle, presented as a spheroid entity with a firm, granular consistency and poorly delineated margins. A defect measuring 4 x 6 cm was noted in the abdominal wall because we tried to get some margins all around, posing a significant risk for herniation. This anterior abdominal wall defect, consequent to the resection of the rectus muscle, was reconstructed using a 6 x 11 cm (Figure 3) polypropylene mesh. We chose the sublay technique for the reconstruction of the abdominal wall, with the mesh put behind the rectus muscle, accompanied by the placement of subcutaneous drains.

**Figure 4:**
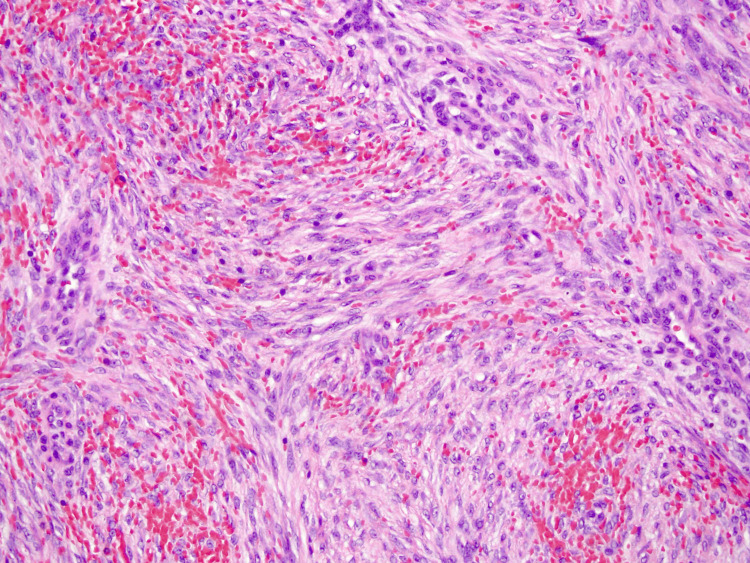
Desmoid tumor spindle cells were sizable, uniform, and immature, with unclear, plain nuclei

Histopathological examination substantiated the diagnosis of a desmoid tumor with clear surgical margins. The analysis revealed a typical pattern of fibroblast and myofibroblast cells. Characteristically, the spindle cells were expansive, uniform, and immature, possessing nuclei that were subtle and nondescript (Figure 4). Following a three-day hospitalization devoid of postoperative complications, the patient was discharged. A six-month follow-up indicated no evidence of tumor reappearance, and the patient underwent a colon endoscopy without any polyps or tumors. The patient's condition remained favorable.

## Discussion

Desmoid tumors are categorized into three distinct types: intra-abdominal, abdominal wall-associated, and extra-abdominal. Despite the varied locations, the histological characteristics of these tumors remain consistent. A majority, approximately 60%, of desmoid tumors are situated extra-abdominally, while 15% manifest within the abdominal cavity. The remaining 25% are localized to the abdominal wall [5]. The distribution of extra-abdominal desmoid tumors is extensive, with a frequent occurrence in the shoulder girdle, lower extremities, and trunk.

The majority of intra-abdominal desmoid tumors are diagnosed in individuals with familial adenomatous polyposis. The concurrent presentation of these two conditions is frequently referred to as Gardner syndrome, which manifests in approximately 2-5% of affected patients [6].

Numerous case reports within the scientific literature have established a definitive correlation between the emergence of abdominal wall desmoid tumors and the gestational as well as postpartum phases [7]. Notably, the preponderance of such cases involves the development of desmoid tumors within the abdominal wall of women who have recently given birth, with a particular propensity for the right rectus abdominis muscle, analogous to the scenario presented herein.

Possible differential diagnoses for this patient include inflammation, endometriosis, and hematoma. The patient's clinical history and physical and laboratory tests revealed no abnormalities, which is frequently useful in reducing differential diagnoses. Palpation or ultrasonography cannot provide a reliable clinical diagnosis of the tumor's origin or differentiate it from endometriosis. The tumor's intramuscular position in ultrasonography indicates a desmoid tumor. In almost every case, desmoids are found near a muscle aponeurosis. Endometriotic tumors of the abdominal wall are most commonly encountered in the subcutaneous region. Possible markers for endometriotic tumors include the relationship between the patient's menstruation and tumor discomfort, as well as the relationship between the tumor volume and menstruation. In the current literature, only 18 case reports concerning endometriosis solely localized in the rectus muscle sheath are available [8].

The precise pathogenesis of desmoid tumors, inclusive of those associated with pregnancy, remains elusive. Hypotheses suggest that hormonal and immunological alterations during pregnancy or the postpartum period may play a significant role. Additionally, the mechanical stress exerted by an enlarging uterus is considered a potential contributing factor to the development of desmoid tumors [9].

A core needle or incisional biopsy is required for an accurate diagnosis prior to surgery. The preferred diagnostic test is an excisional biopsy of the tumor. Before proceeding with a surgical excision, consider obtaining a fine-needle aspiration biopsy specimen. Fine-needle aspiration is relatively reliable for determining the benign nature of desmoids. Occasional over- and under-diagnosis of cancer can occur; however, a core needle biopsy appears to be more trustworthy. Electron microscopy may be used. The appearance of fine-needle aspiration smears of desmoids was consistent, but the yield varied greatly, with some smears containing little material and others being densely packed with cells. Core needle biopsies revealed considerable collagen-producing fascicular spindle cell proliferation among fibroblastic and myofibroblastic cells, with no cytologic atypia. Thin-walled ectatic vessels indicative of desmoid were frequently found. Electron microscopy reveals spindle cells in desmoid tumors to be myofibroblasts. These data suggest an aberrant multiplication of myofibroblasts, which typically vanish after wound healing. The aforementioned characteristics are in contrast to those found in fibrosarcoma, which has more mitotic activity, a higher nuclear-to-cytoplasm ratio, greater vascularity, less collagen synthesis, and fewer immune cells [10].

In histopathological terms, desmoid tumors are composed of spindle-shaped fibroblasts and myofibroblasts. These cells are distinguished by their slender, tapering cytoplasmic extensions; elongated nuclei with a vesicular appearance and conventional morphology; and the presence of several diminutive nucleoli. The cellular arrangement is linear, with individual cells being encased and demarcated by an abundant collagenous matrix [1].

The diagnosis of desmoid tumors relies heavily on multimodality imaging, which includes ultrasound, CT, and MRI. The positive detection of a desmoid tumor is not always easy; it is frequently misdiagnosed with parietal endometriosis due to similar clinical indications and nonspecific imaging studies. Ultrasound can help with diagnosis by finding the tumor: an intramuscular or aponeurotic location typically implies a desmoid, but a subcutaneous topography may indicate an endometrioma. The major ultrasound features of desmoid tumors are oval form, imprecisely delineated edges, and various hypo- and hyperechogenic regions [11].

Through ultrasonographic evaluation, desmoid tumors are observed as lesions with distinct contours exhibiting a spectrum of echogenicity. The peripheral margins of these tumors can sometimes manifest as indistinct or irregular [1].

CT is routinely used to image desmoid tumors, and it is especially useful for intraabdominal lesions. The amount of collagen and myxoid content may determine the CT look of the tumor, as it does with ultrasonography. Myxoid tumors are often hypodense in comparison to skeletal muscle, but collagenous and fibrotic components might be isodense or hyperdense [12,13]. Desmoid tumors normally improve after intravenous contrast injection; however, the degree of improvement is usually mild to moderate due to the presence of variable myxoid and collagenous material inside the tumor [14,15]. Necrosis is usually absent [16]. CT scans can provide essential information for treatment planning, such as the tumor's connection to main vessels and surrounding organs.

Because of its superior soft tissue resolution, MRI is extremely useful for imaging desmoid tumors. Desmoid tumors' MRI features are determined by their histological components [13,17-19]. Fibrotic and collagenous portions of desmoid tumors typically have low signal intensity on T2-weighted sequences and show mild-to-moderate enhancement, particularly on delayed phase post-contrast enhanced images; in contrast, prominent cellular stroma and myxoid matrix in desmoid tumors show heterogeneous T2-hyperintense areas and moderate-to-intense enhancement following intravenous contrast administration. The presence of linear, non-enhancing, T1- and T2-hypointense bands within the tumor (band sign) has been identified as a distinctive MRI feature in 60-90% of desmoid tumors [17]. However, it should be emphasized that the band sign is not specific to desmoid tumors, as other musculoskeletal soft tissue cancers, such as malignant fibrous histiocytoma, may exhibit this imaging feature [17,18]. The fascial tail sign is the presence of a linear infiltrative boundary extending from the tumor along the fascial plane, which can be found in up to 83% of desmoid tumors [17].

CT and MRI features can help in diagnosis and patient management. The tumor's relationship to the surrounding structures (particularly major neurovascular structures and essential organs) should be thoroughly assessed and recorded, as this can help determine the feasibility of surgery.

The surgical management of desmoid-type fibromatosis presents a considerable challenge to medical practitioners due to its infrequent occurrence and propensity for invasive growth into adjacent healthy tissues. Therapeutic strategies for desmoid tumors are tailored to the specific clinical scenario of each patient. For desmoid tumors that are stable and asymptomatic, a conservative approach involving periodic monitoring through imaging at intervals of three to six months is often adopted. Therapeutic intervention is imperative for individuals exhibiting symptoms, particularly in cases where the tumor exerts a mass effect on vital anatomical structures. Surgical excision constitutes the primary modality of treatment for patients diagnosed with extra-abdominal desmoid tumors, which encompass tumors of the abdominal wall as exemplified in the present scenario. While certain scholars assert that tumor recurrence is not necessarily linked to the achievement of negative surgical margins, the prevailing consensus among researchers is to advocate for surgical resection with an ample margin of healthy tissue to reduce the likelihood of local recurrence. Furthermore, a retrospective examination of 189 cases of desmoid tumor patients indicated that the existence of residual tumor cells at the microscopic surgical margin constituted the most critical factor influencing the recurrence rate in individuals who underwent surgical treatment. Consequently, for substantial desmoid tumors, it is advisable to contemplate reconstructive techniques, including the transposition of aponeurotic-muscular flaps or the employment of prosthetic mesh, as part of the preoperative planning process [8].

## Conclusions

In clinical practice, the differential diagnosis of a desmoid tumor ought to be given substantial consideration in female patients with a history of recent parturition who present with an abdominal wall mass, despite its rarity. CT scanning is regarded as the definitive modality for precise identification and differentiation from other etiologies. A core needle aspiration biopsy specimen may be considered before going for surgical excision and recognizing the benign nature of desmoids. The surgical intervention for desmoid-type fibromatosis and subsequent reconstruction of the abdominal wall defect is both viable and presents a significant challenge.
